# Low-fluence vs. standard fluence hair removal: A contralateral control non-inferiority study

**DOI:** 10.3109/14764172.2011.634421

**Published:** 2012-01-31

**Authors:** Shlomit Halachmi, Moshe Lapidoth

**Affiliations:** 1 Laser Unit, Department of Dermatology, Rabin Medical Center, Petach Tikva, Israel; 2 Sackler School of Medicine, Tel Aviv University, Tel Aviv, Israel

**Keywords:** fluence, hair removal, laser, low fluence, selective photothermolysis

## Abstract

**Introduction:**

Laser hair removal at lower fluences, delivered under certain conditions, may retain the efficacy of high-fluence lasers while improving tolerability. We performed a pilot study comparing the efficacy, safety and tolerability of laser hair removal using traditional settings compared to lower fluences, delivered from a larger handpiece and under vacuum.

**Material and methods:**

Fourteen healthy participants underwent 5 axillary hair removal treatments with an 800 nm diode laser at 1-month intervals, with follow-up 1 and 3 months after the 5th treatment. In all patients, one side was treated with standard parameters using a 9 × 9 mm chilled tip and gel, while the contralateral side was treated using a 22 × 35 mm vacuum-assisted handpiece at fluences up to 12 J/cm^2^. Follow-up assessments were performed after each treatment and at each follow-up visit, and included photography and questionnaires.

**Results:**

Eleven participants completed the study and follow-up. All experienced significant hair removal in all treated areas. At the 3-month follow-up visit, the high-fluence and low-fluence treated axillae demonstrated comparable hair reduction. Participants found the lower fluence treatments to be more tolerable. No adverse events were reported.

**Conclusion:**

Lower fluence diode laser, delivered under conditions of vacuum and using larger spot sizes, can provide significant hair reduction.

## Introduction

The use of selective photothermolysis in laser hair removal was first demonstrated in 1996, with targeted heating of melanin in the follicular unit by a 694-nm ruby laser (1,2). Since the theory is predicated on the preferential absorption of light at particular wavelengths by melanin, other wavelengths with desirable melanin absorption curves have been employed as well, including diode, alexandrite and Nd:YAG (3–9). In keeping with the theory of selective photothermolysis, the treatment parameters that have become standard aim for a high peak fluence and short pulse duration, to maximize efficacy and selectivity. While higher fluences are reported to induce better hair reduction, the use of higher fluence is associated with greater pain and increased risk of certain adverse events, primarily thermal burns, blisters, pigmentary changes and scarring (8,10). However, lower fluences of laser have been demonstrated to induce damage in the follicular structure (11–13). Given the need to balance efficacy with safety and tolerability, approaches that reduce fluence but remain within the effective treatment range may provide clinical benefit.

The factors that contribute to efficacy arise from the parameters perceived by the hair follicle, e.g. the actual temperature rise at the follicle. As in other uses of lasers in cutaneous treatments, in addition to fluence, the variables that are adjusted to match the target are the pulse duration and the spot size. The pulse duration is adjusted to maximize the heating of the target relative to surrounding structures, as proposed by the theory of selective photothermolysis. The spot size is chosen with multiple criteria: to match the size of the treatment area so as to minimize treatment time, and to achieve variable depth.

**Figure 1:**
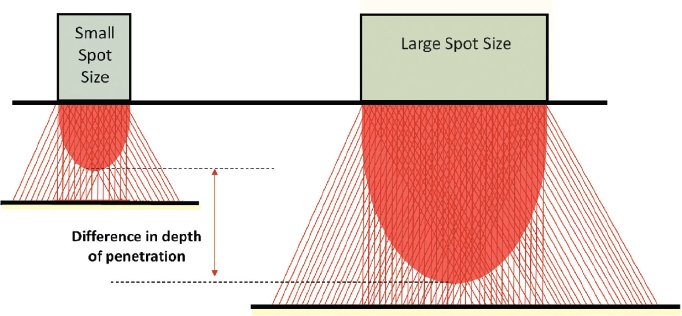
Effect of dermal scatter on beam propagation.

With these factors in mind, optimal heating of the hair follicle at the level of the deep dermis can be achieved by altering not only fluence and pulse duration, but also by adjusting the spot size. It is known that small spot sizes require higher fluences to heat dermal targets effectively. Studies have shown that larger spot sizes are more effective for laser assisted hair removal (14–16). The effect of spot size on the depth of laser penetration is explained at least in part by the phenomenon of dermal scattering (Figure 1). As a result, as spot size increases, the light penetrates deeper. Consequently, a larger spot size allows more effective heating, and conversely deeper heating can be achieved with lower fluences when delivered with a larger spot size (17).

**Figure 3:**
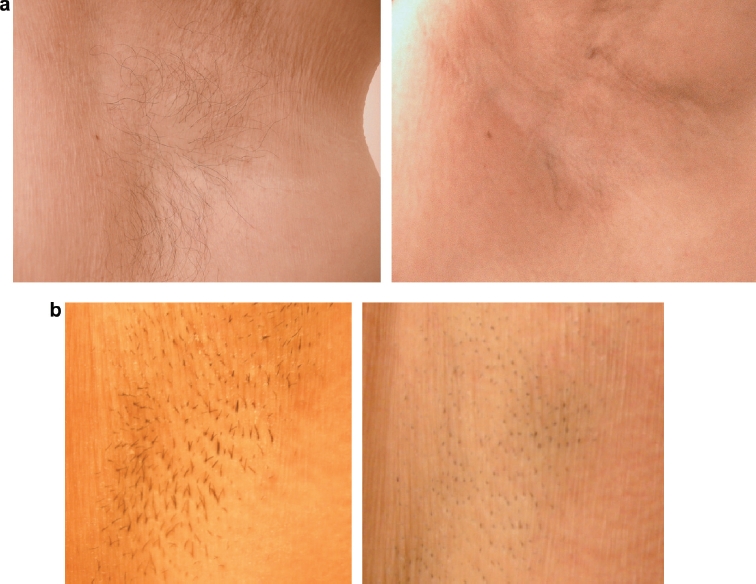
a. 60-year- old female, before and 3 months after 5 treatments with the HS handpiece. b. 28–year-old female, before and 3 months after 5 treatments with the HS handpiece.

This theory is the basis for the use of a very large spot size for low-fluence hair removal. Specifically, the use of a very large spot size should allow efficient temperature rise at the depth of the hair follicle with lower fluences. The LightSheer 800 nm diode laser (Lumenis Ltd.), which was introduced in 1998, has recently been expanded in the Duet model to incorporate a second, larger ‘high speed’ (HS) handpiece of 23 × 35 mm, which operates at fluences up to 12 J/cm^2^ (Figure 3). The handpiece operates by drawing the skin into a gold-plated chamber using vacuum. The laser light is then emitted from diodes at the top of the concave handpiece, and any reflected light that reaches the gold-plated sidewalls of the handpiece chamber is redirected to the skin.

**Figure 2:**
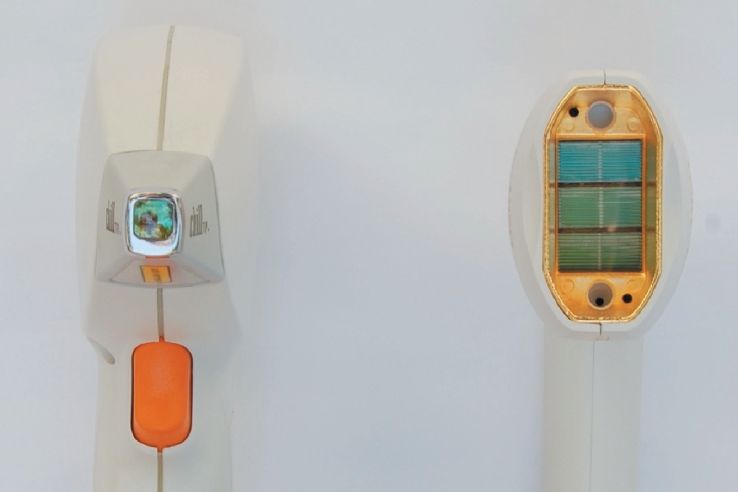
ET and HS handpieces.

In order to assess the effectiveness of hair removal by the large HS (high speed) handpiece, we performed a non-inferiority assessment of the traditional 9 × 9 mm handpiece to the 23 × 35 mm handpiece in a head-to-head contralateral control study (Figure 2). Fourteen participants underwent five treatments each, in which parallel treatments were administered to the axillae. In each treatment, one side consistently underwent treatment with the 9 × 9 mm ET handpiece at traditional high fiuence settings, while the contralateral side was treated with the 23 × 35 mm HS handpiece.

## Material and methods

All participants signed informed consent prior to treatment. All procedures conformed to the guidelines set forth by the Declaration of Helsinki. Females aged 18–65, in good general health, with Fitzpatrick skin types I-IV and brown or black hair were eligible for the study. Participants were excluded for pregnancy, active skin disease in the treatment area, or prior laser hair removal procedures in the treatment area. Participants underwent 5-monthly laser hair removal treatments with the LightSheer Duet 800 nm diode laser (Lumenis Ltd, Israel). Photography was performed prior to each treatment and 1 and 3 months after the last treatment. The right axilla was treated with the ET handpiece, using parameters of 25–35 J/cm^2^, pulse duration 30 ms, with contact cooling and gel. The left axilla was treated with the HS handpiece, at triple pulses of 4.5–6 J/cm^2^ with pulse duration of 30 ms and low vacuum for the first three treatments, and at single pulses of 11–12 J/cm^2^ with pulse duration of 60 ms and low vacuum for the subsequent two treatments. Participants completed feedback questionnaires after the 3rd and 5th treatments and at the 1 and 3-month follow up visits. The questionnaires assessed preference of the HS vs. the ET. Specifically, the questions inquired which handpiece was preferred, satisfaction with the HS on a six-point scale (extremely satisfied, very satisfied, somewhat satisfied, somewhat dissatisfied, very dissatisfied, extremely dissatisfied), likelihood to return for additional treatments with the LightSheer Duet using the HS handpiece on another body area (extremely likely, very likely, somewhat likely, somewhat unlikely, very unlikely, extremely unlikely), and the likelihood of recommending to others (extremely likely, very likely, somewhat likely, somewhat unlikely, very unlikely, extremely unlikely).

## Results

Fourteen participants were enrolled. The mean age was 32 (17–61). Fitzpatrick skin types represented were II (3, 21%), III (9, 64%), and IV (2, 14%). Fourteen percent had coarse hair, 57% had medium hair and 29% had relatively fine hair. Eleven participants completed all treatments and follow-up visits; those who did not complete the study left due to pregnancy (one participant) or scheduling difficulties (two participants).

Treatment was well-tolerated by all participants, with no adverse events in either the HS or ET treatment areas. Reduced hair growth was observed in all patients in both the ET and HS treated areas at the 1-month and 3-month follow-up visit. After five treatments, minimal to no differences were visibly appreciable between the ET and HS treated sides (Figure 3). The equivalence in response was noted after treatment 5, slightly less so after treatment 4. Prior to treatment 4, there was a slightly longer time to hair regrowth in the ET treated areas.

At conclusion of the treatment, 73% of participants preferred the HS handpiece to the ET handpiece (Table I). In rating discomfort, 67.8% of participant responses described discomfort with the HS as none, little or moderate, compared to 58.1% for the ET handpiece. Ten of the eleven participants who completed treatment (91 %) were satisfied with the HS treatment; 83% replied that they would continue treatment of other body areas after the study, at their own cost, and 87% would recommend the treatment to others (Tables II, III, IV).

**Table I:** Handpiece preference: HS vs. ET.

	Handpiece preference (%)
HS	73
ET	27
No preference	0
	*100*

(11 respondents).

**Table II:** Satisfaction score: HS.

	Percent (%)
Satisfied (score 1–4)	91
Dissatisfied (score 5–6)	9
	*100*

(11 respondents).

**Table III:** Likelihood of seeking additional HS treatment after conclusion of study.

	Percent (%)
Likely to seek more	82
Unlikely to seek more	18
	*100*

(11 respondents).

**Table IV:** Likelihood of recommending HS treatment.

	Percent (%)
Likely to recommend	87
Unlikely to recommend	13
	*100*

(11 respondents).

## Discussion

Laser hair removal has a long track record of safety and efficacy, but it still suffers from long treatment times and the risk of dose-related adverse events. Large spot sizes reduce the treatment time, and they are expected to allow effective hair removal with lower fluences than small spot sizes. To evaluate the safety and efficacy of low-fluence, large spot size treatments in laser hair removal, we compared it to traditional diode high-fluence treatments in a head-to-head non-inferiority study in axillary laser hair removal. The major benefit of lower fluence treatments is the reduced risk of adverse events. No adverse events were noted in the HS-treated axillae, and participants preferred the HS treatment for discomfort, the most common unwanted effect of laser hair removal.

The results demonstrate that both traditional (ET handpiece) and low-fluence, large spot, high speed (HS) provide significant hair reduction with 3-months follow-up after five treatments. After five treatments, no difference could be detected between the ET- and HS- treated axillae. It should be noted that after the first three treatments, a mild difference was notable, with slightly more rapid regrowth of hair in the HS-treated axillae relative to the ET-treated axillae. Providers and patients who are accustomed to long periods without hair growth after traditional diode laser treatments should be aware of the relatively faster recurrence of hair in HS-treated areas, but should also be aware that the discrepancy is undetectable after five treatments.

The non-inferiority demonstrated in this study, namely that 12 J large spot size treatments can provide equivalent hair removal to 25–30 J small spot size treatments after five treatment sessions, is likely multi-factorial. The large spot size, with its greater ability to heat the deeper dermis, is likely to play a key role in this effect. One might ask why intense pulse light (IPL) devices, which also have large spot sizes, do not exhibit the same efficacy with low fluences. A plausible explanation is that in IPL the energy is distributed over a wide range of light, and because of that there is not sufficient energy in the wavelengths critical for hair removal to allow sufficient heating at the level of the follicle. Furthermore, the light in IPL is non-coherent, and the large spot size may not overcome the dermal scatter. An alternate explanation may be that the three-dimensional geometry of the skin, as it is raised into the handpiece of the HS by the vacuum, together with the gold-plated chamber, optimizes the delivery of light in a way that improves on the delivery over a flat interface, such as the contact of a crystaltipped IPL and the skin. Finally, it is also possible that the vacuum mechanism of the HS handpiece increases temporarily the amount of hemoglobin in the treatment area. It has been proposed that the effect of hair removal is vascular in nature, with the damage occurring at least in part due to heating of the vessels which supply the pilosebaceous unit (18). This would allow a benefit to low fluences applied with vacuum over low fluence treatments in which the handpiece is pressed onto the skin, thereby compressing the blood vessels.

## References

[1] Anderson RR, Parrish JA (1983). Selective photothermolysis: precise microsurgery by selective absorption of pulsed radiation. Science.

[2] Grossman MC, Dierickx C, Farinelli W, Flotte T, Anderson RR (1996). Damage to hair follicles by normal-mode ruby laser pulses. J Am Acad Dermatol.

[3] Finkel B, Eliezri YD, Waldman A, Slatkine M (1997). Pulsed alexandrite laser technology for noninvasive hair removal. J Clin Laser Med Surg.

[4] Lask G, Elman M, Slatkine M, Waldman A, Rozenberg Z (1997). Laser-assisted hair removal by selective photothermolysis. Preliminary results. Dermatol Surg.

[5] Bencini PL, Luci A, Galimberti M, Ferranti G (1999). Long-term epilation with long-pulsed neodimium:YAG laser. Dermatol Surg.

[6] Ross EV, Ladin Z, Kreindel M, Dierickx C (1999). Theoretical considerations in laser hair removal. Dermatol Clin.

[7] Williams RM, Gladstone HB, Moy RL (1999). Hair removal using an 810 nm gallium aluminum arsenide semiconductor diode laser: A preliminary study. Dermatol Surg.

[8] Campos VB, Dierickx CC, Farinelli WA, Lin TY, Manuskiatti W, Anderson RR (2000). Hair removal with an 800-nm pulsed diode laser. J Am Acad Dermatol.

[9] Dierickx CC (2000). Hair removal by lasers and intense pulsed light sources. Semin Cutan Med Surg.

[10] Campos VB, Dierickx CC, Farinelli WA, Lin TY, Manuskiatti W, Anderson RR (2000). Ruby laser hair removal: Evaluation of long-term efficacy and side effects. Lasers Surg Med.

[11] Liew SH, Ladhani K, Grobbelaar AO, Gault DT, Sanders R (1999). Ruby laser-assisted hair removal success in relation to anatomic factors and melanin content of hair follicles. Plast Reconstr Surg.

[12] Trelles MA, Urdiales F, Al-Zarouni M (2010). Hair structures are effectively altered during 810 nm diode laser hair epilation at low fluences. J Dermatolog Treat.

[13] Kato T, Omi T, Naito Z, Hirai T, Kawana S (2004). Histological hair removal study by ruby or alexandrite laser with comparative study on the effects of wavelength and fluence. J Cosmet Laser Ther.

[14] Nouri K, Chen H, Saghari S, Ricotti CA (2004). Comparing 18-versus 12-mm spot size in hair removal using a gentlease 755-nm alexandrite laser. Dermatol Surg.

[15] Raff K, Landthaler M, Hohenleutner U (2004). Optimizing treatment parameters for hair removal using long-pulsed Nd: YAG-lasers. Lasers Med Sci.

[16] Zhao Z-Q, Fairchild PW (1998). Dependence of light transmission through human skin on incident beam diameter at different wavelengths. Proc. SPIE 3254,.

[17] Kaminer MS, Dover JS, Arndt KA (2002). Atlas of cosmetic surgery.

[18] Adrian RM (2000). Vascular mechanisms in laser hair removal. J Cutan Laser Ther.

